# CBMAR: a comprehensive β-lactamase molecular annotation resource

**DOI:** 10.1093/database/bau111

**Published:** 2014-12-03

**Authors:** Abhishikha Srivastava, Neelja Singhal, Manisha Goel, Jugsharan Singh Virdi, Manish Kumar

**Affiliations:** ^1^Department of Biophysics and ^2^Department of Microbiology, University of Delhi South Campus, Benito Juarez Road, New Delhi 110021, India

## Abstract

β-Lactam antibiotics are among the most widely used antibiotics against microbial pathogens. However, enzymatic hydrolysis of these antibiotics by bacterial β-lactamases is increasingly compromising their efficiency. Although new generation β-lactam antibiotics have been developed to combat antibiotic resistance, β-lactamases have also evolved along with the new variants of the substrate. A strong selection pressure from the newer generation of β-lactam antibiotics has resulted in evolution of different families within each class of β-lactamase. To facilitate detailed characterization of different families of β-lactamases, we have created a database, CBMAR, which facilitates comprehensive molecular annotation and discovery of novel β-lactamases. As against the limited scope of other existing similar databases, CBMAR provides information useful for molecular and biochemical characterization of each family of β-lactamase. The basic architecture of CBMAR is based on Ambler classification, which divides β-lactamases as serine (Classes A, C and D) and metallo-β-lactamases (Class B). Each class is further divided into several families on the basis of their hydrolytic character. In CBMAR, each family is annotated with (i) sequence variability, (ii) antibiotic resistance profile, (iii) inhibitor susceptibility, (iv) active site, (v) family fingerprints, (vi) mutational profile, (vii) variants, (viii) gene location, (ix) phylogenetic tree and several other features. Each entry also has external links to the relevant protein/nucleotide sequence and structure databases. The database also supports sequence similarity searches using BLAST and assigns a new β-lactamase protein to its respective family on the basis of family-specific fingerprint.

**Database URL:** http://14.139.227.92/mkumar/lactamasedb

## Introduction

β-Lactam antibiotics are the most widely used anti-microbial agents. Over the years, continuous and indiscriminate use of antibiotics has led to the evolution of resistance against them. There are many ways through which a pathogen can evade the action of antibiotics but the main cause of resistance against β-lactam antibiotics is the irreversible hydrolysis of the amide bond of the β-lactam ring, resulting in a biologically inactive product (1, 2).

β-Lactamase enzymes encompass a large and diverse group of enzymes which can be classified on the basis of primary structure-Ambler classification (3) or on the basis of their characteristics-Bush classification (4). Ambler initially classified β-lactamases into two classes, A and B. Class A enzymes were serine β-lactamases whereas Class B were zinc containing metallo-β-lactamases. Later, two new classes of serine β-lactamase were discovered that shared a little sequence similarity to the known Class A enzymes and were designated as Classes C and D (5, 6). Serine β-lactamases hydrolyze their substrate by forming a serine bound acyl intermediate, whereas metallo-β-lactamases utilize active site zinc ion to facilitate β-lactam hydrolysis and the catalysis does not require formation of a covalent bond (4).

To tide over increasing β-lactamases-mediated resistance, newer generation β-lactam antibiotics were discovered. Although the newer generation antibiotics are more effective than their predecessors, they have exerted a stronger selection pressure on β-lactamases resulting in evolution of newer variants of β-lactamases. These newer variants of β-lactamases are denoted as extended spectrum β-lactamases. The problem of antibiotic resistance cannot be addressed until we gain a fair understanding of β-lactamase sequences and relationship between their structure and function. Also analysis of individual mutations leading to expansion of hydrolytic profile can help in prediction of the future course of evolution (7–10). The detailed analysis of this is not possible until all information is arranged systematically at one place.

**Figure 1.**
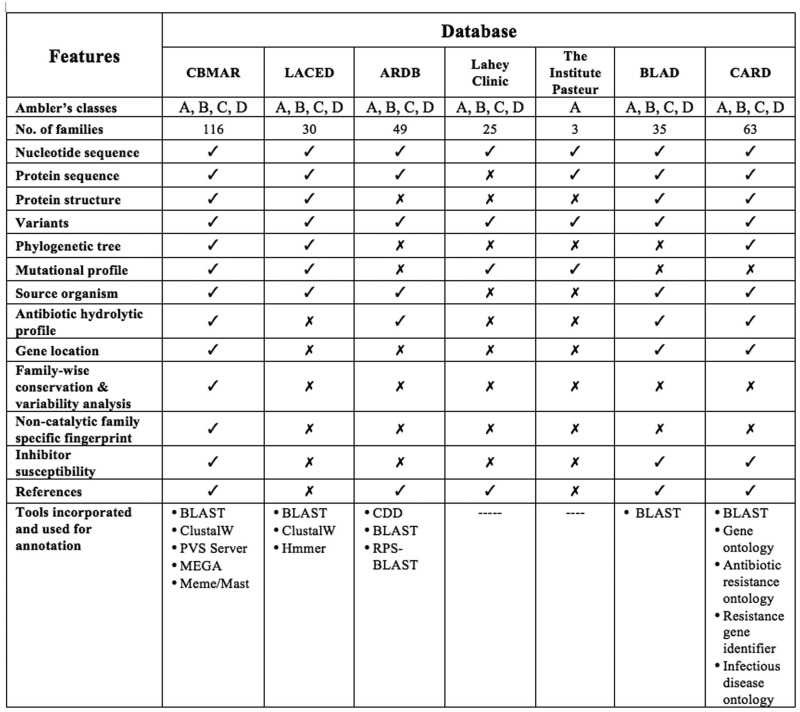
Comparison of CBMAR with existing β-lactamase information resources.

A few efforts have been made in the past to establish knowledge-banks of β-lactamase-mediated antibiotic resistance, for example, (i) The Lahey clinic database (www.lahey.org/Studies/), (ii) Antibiotic Resistance Genes Database (ARDB) (11), (iii) Lactamase Engineering Database (LacED) (12), (iv) The Comprehensive Antibiotic Resistance Database (CARD) (13), (v) The Institut Pasteur Database and (vi) BLAD: ‘A comprehensive database of widely circulated β-lactamases’ (14). Although the information content of these databases was useful and played an important role in understanding antibiotic resistance, none of the above-mentioned databases provided comprehensive information at one place. The Lahey Clinic database contains comprehensive collection of serine β-lactamases. It also attempts to standardize the nomenclature for β-lactamase genes and the amino acid sequences, but only of TEM, SHV, OXA extended spectrum and some other inhibitor resistant enzymes. ARDB contains information about the genes mediating resistance to only a few β-lactam antibiotics like vancomycin and tetracycline; also as per its website, it was last updated in July 2009. The LacED provides information specifically only for mutations, sequences and structures of TEM and SHV β-lactamases. The Institute Pasteur database includes protein variation information for OXY, OKP and LEN β-lactamases only. Although the CARD database is the most extensive and provides all relevant factors responsible for pathogen antibiotic resistance, it contains a limited number of β-lactamase families (63 in total). Also, as the data in the CARD database are classified and organized using Sequence Ontology (13), a series of navigations are required to reach a specific class of proteins, like β-lactamases. Further, it also lacks the information often vital for a laboratory working on β-lactamases, like its mutational profile, inhibitor profile and regions of high variability or conservation. The recently published database BLAD (14) contains information only about the ‘widely circulated β-lactamases’. In addition to these specialized resources, β-lactam antibiotic resistance information can also be extracted from popular molecular biology databases like GenBank (15) and UniProt (16). Although GenBank and UniProt are the primary source of data for any specialized database, they lack the information often crucial for specialized research like antibiotic resistance. Therefore, a centralized and updated resource can serve the purpose. Moreover, none of the databases mentioned above provide any biochemical information viz. catalytic residues, inhibitor susceptibility, variability and mutational profile, which might help in understanding the catalytic dynamics of newly discovered β-lactamases. These databases offer limited resources and data to integrate molecular information ranging from genes and their products, to antibiotics and their associated literature (Figure 1).

**Figure 2.**
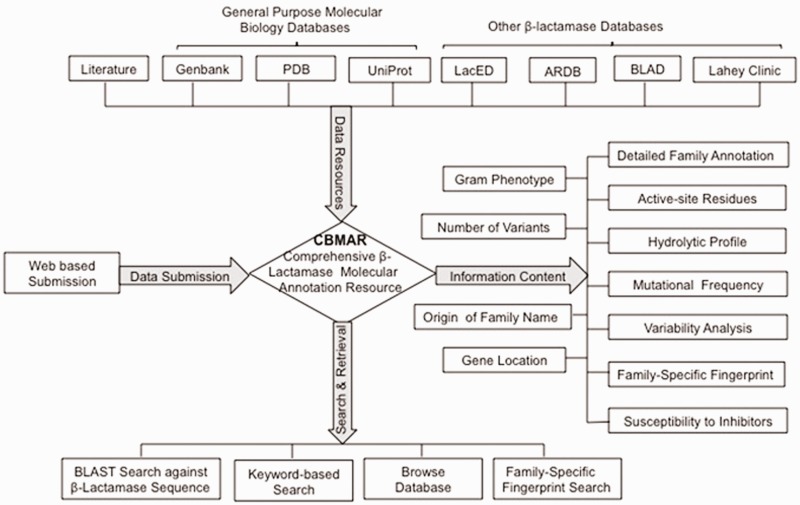
A schematic representation of the CBMAR database.

Here, we describe a new database—CBMAR, which provides information about molecular and biochemical functionality of β-lactamases. The basic architecture of CBMAR is based on the Ambler classification. Each class is further divided into several families on the basis of their antibiotic hydrolytic profile and sequence similarity. Each family is annotated with (i) origin of the name of the family, (ii) genus/genera in which a particular family of β-lactamase was reported, (iii) genomic location (chromosomal/plasmidic), (iv) antibiotic resistance profile, (v) inhibitor susceptibility, (vi) active site, (vii) family-specific fingerprints, (viii) mutational profile, (ix) phylogenetic tree and (x) names of variants (Figure 2). Each family is also externally linked to other sequence and structure databases. The database also supports sequence similarity searches using BLAST and search for family-specific fingerprints using MAST (17). It is publicly accessible at http://14.139.227.92/mkumar/lactamasedb and would be updated regularly.

## CBMAR Data content and overview

### Data collection

We used protein sequences from UniProt (16) as a seed to retrieve β-lactamases from existing primary molecular biology databases. Initially protein sequences were retrieved from UniProt using ‘beta-lactamase*’ and ‘bla*’ genes as keyword. Using the sequences obtained from UniProt, we carried out BLAST-based database searches against UniProt and NCBI NR databases with threshold *e*-values of 10^−^^4^ to ‘fish out’ any missing proteins. Corresponding gene and nucleotide sequence information of each protein was retrieved from the NCBI GenBank Database. The integrity and quality of gene sequences was validated by aligning proteins with corresponding translated gene sequence. The information about Gram phenotype, sequence variability, antibiotic resistance profile, inhibitor susceptibility, active site, mutational profile, variants and genomic location was collected from the published literature and other databases. The database presently contains manual annotations. Whenever a new β-lactamase will be added to the database, all the programs would have to be rerun to accommodate the new entry, which is not a trivial work. In future, the curator plans to automate all annotation pipelines so that once a newly reported β-lactamase gene passes the initial quality check, annotation can be done automatically. Number of variants and proteins in each family along with the Gram phenotype and location of gene in the host organism is given in Supplementary Table S1.

### Classification of β-lactamases

**Figure 3.**
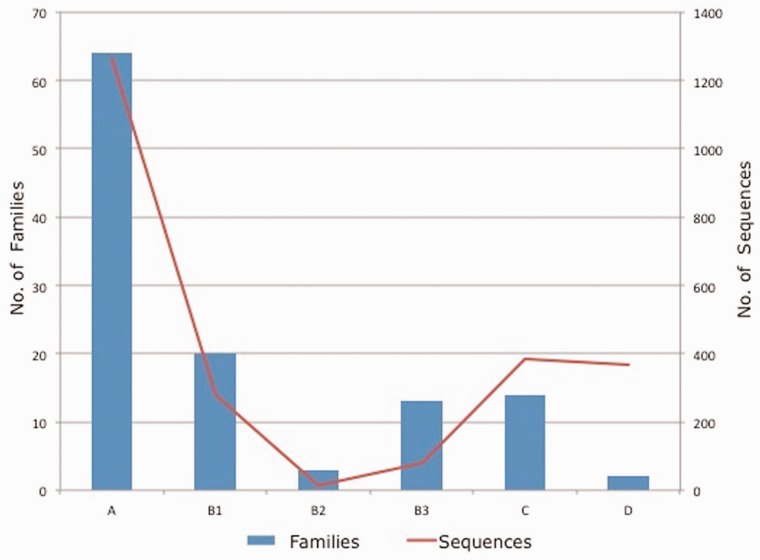
Class/subclass wise statistics of the annotated families and sequences present in CBMAR.

Each protein included in the database was assigned to an Ambler class and a specific family. In order to verify family classification, multiple sequence alignment was also built using CLUSTAL W 2.1 (18) to reconfirm the family assignment. Class B β-lactamases were further classified into three subclasses: B1, B2 and B3 (19). Figure 3 shows the data statistics of number of families and sequences present in each class/subclass of β-lactamase contained in the CBMAR.

### Database architecture and web interface

CBMAR is built on an Apache HTTP Server 2.2.17 with MySQL Ver 14.14 and hosted on Ubuntu 11.04 Linux platform. All scripts have been written using programming language Perl. The web-interface was built using HTML and CGI Perl was used to communicate between HTTP server and web-interface. DBD::mysql was used as the Perl interface driver for MySQL.

## Utilities and discussion

### Data accessibility

**Figure 4.**
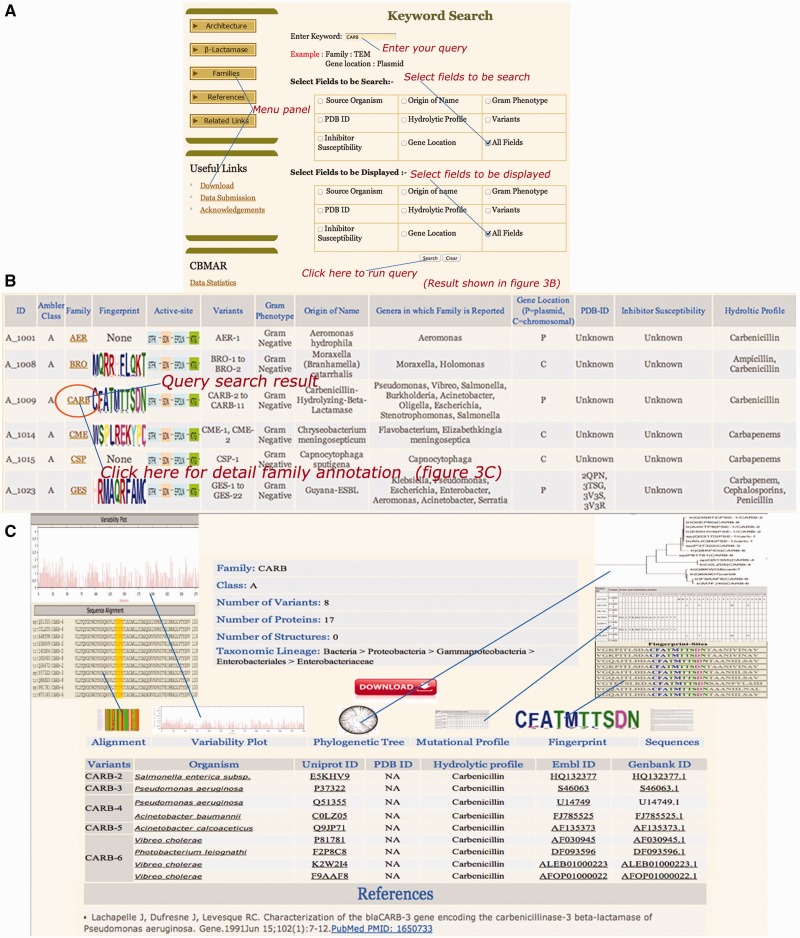
Snapshots of ‘CARB’ keyword search (A), result page (B) and family details (C). The search interface allows for the query search over one or multiple fields. Similarly, the display of result can also be limited to one or multiple fields. The result displays the search results along with appropriate hyperlinks for detailed family annotation, fingerprint and active site residues.

The CBMAR provides interactive access to the data and the user can connect to the database using any web browser. Figure 4A shows a snapshot of the user interface to browse or search the database. A user can retrieve information from CBMAR in the following ways:

#### Keyword search

The keyword search option allows the user to perform search on any/all fields of the database (Figure 4A). In response to the keyword search, the database returns a table of related records, which lists the families containing the matching query word. Along with each family, information about its Ambler class, family-specific fingerprint, active-site consensus sequences, origin of the name of the family, genus/genera in which this family of β-lactamase was reported, location of gene in the genome (plasmid/chromosome), PDB IDs, inhibitors, Gram phenotypes of the organisms in which family members of a β-lactamase were reported, variants and hydrolytic profile is also displayed. An example of keyword search for ‘CARB’ in all fields is shown in Figure 4A. The option of display was set to ‘all fields’. The output/result of the search is shown in Figure 4B. The fingerprint images are clickable links and can display detailed information pertaining to the corresponding fingerprints. It includes the sequence of the fingerprint, its location within the sequence and details of the regions harboring these fingerprints. For each family, in addition to the fingerprint, information about catalytic residues and other family-specific details can also be accessed by clicking the family names as shown in Figure 4C. Similar to the fingerprints, the active-site images are also hyperlinked to the sequences of corresponding family, highlighting the sequence coordinates of active site in the protein sequence.

#### ‘CBMAR BLAST’ search against β-lactamase sequences

The BLAST tool has been integrated into CBMAR to search the homologs of a user-provided query sequence from the sequences available in the database. This utility may be useful for initial characterization of the newly discovered and previously unannotated sequences and in identification of homologous β-lactamase sequences present in the database. The search result is the standard BLAST output, arranged in the ascending order of e-values.

#### ‘LactFp’-based family-specific fingerprint search

CBMAR also integrates ‘LactFp’ webserver (20) to facilitate quick identification of family-specific fingerprints, unique to a particular β-lactamase family. The user can submit a protein sequence in order to predict the β-lactamase family to which the query sequence might belong. The search result is provided in a tabular format containing three columns: (i) protein name, (ii) family-specific fingerprints, (iii) e-value and (iv) β-lactamase family to which the query sequence might belong. These fingerprints are extracted from a dataset of 605 manually curated β-lactamase protein sequences.

### Family-specific information

Ambler classification is the basic skeleton around which the CBMAR database has been built. Protein variants with high similarity were assigned to a single family, which was named according to its characteristics like origin of name, hydrolytic activity toward β-lactam antibiotics, geographical diversity, etc. Homologous families were grouped into different classes based on the Ambler classification scheme: (A–D). Families belonging to Class B were further subdivided into B1, B2 and B3 (19).

Each β-lactamase family has its own detailed description page, which contains short summary related to the corresponding β-lactamase family and other related information describing the number of variants, organisms in which members of a particular β-lactamase family were reported, nucleotide/protein sequences, PDB structures, hydrolytic profile and link to other databases and description. The family-specific description page can be accessed either by clicking the family name in the search output or from the tab ‘Families’ in the main menu. It also includes a ‘References’ section that provides information about research papers, which have described that particular family. Each research paper is also hyperlinked to PubMed (15). Other features like mutational profile, variability plots and multiple sequence alignments are also available in the family page.

#### Family-specific fingerprints

These are conserved patterns present in β-lactamase families, which may act as an unique identification fingerprints for a β-lactamase family (20). They were derived using the pattern-search tool MEME (17) with default parameters of fingerprint width of 10 residues and minimum occurrence of one pattern per sequence. In order to verify the identified signature patterns, we used MAST (17) for cross-validation using UniProt protein database. The CBMAR catalogs a total of 70 fingerprints corresponding to 70 families. A detailed description of family-specific fingerprint identification and validation can be found here (20).

#### Active-site residue annotation

The amino acid residues, which participate in the formation of active site in a β-lactamase family, are also marked. These include residues that directly take part in catalysis and also residues, which only form the cavity but do not participate in catalysis. The active*-*site residues of each family were deduced after a comprehensive survey of the published literature and conservation analysis.

#### Multiple sequence alignment, phylogenetic tree and variability plot

It is assumed that conserved residues are critical for enzyme stability and/or function, wh*ereas* variable residues are assumed to be less critical. We have performed multiple sequence alignment for protein sequences belonging to each β-lactamase family to identify critically conserved residues that might participate in β-lactam hydrolysis under the section ‘Sequence Alignment’. In order to further investigate how frequently and to what degree variable residues are mutated, we have annotated their substitutions under the section ‘Mutational profile’.

The long list of variants in each family of β-lactamase indicates that it is a fast evolving family of proteins. All residues in a protein do not contribute equally to the structure and function. Residues at certain positions are essential and therefore substitutions at these positions may result in a non-functional protein. However, other residue positions might be less important and thus substitutions at these positions will be more frequent. Therefore, identification and discrimination between essential and non-essential residues can play an important role in understanding the molecular detail of β-lactamase-mediated antibiotic resistance. The sequence variability pattern in each family of β-lactamase was generated using the protein variability server (PVS) (21, 22). PVS estimates the variability at each position of multiply aligned protein sequences in terms of Shannon Entropy (23). In variability plot higher the entropy at a position, more variable are the residues at that position whereas low entropy indicates a conserved region. In order to facilitate the visualization of sequence variability analysis *vis-à-vis* whole family, variability plot is also provided along with multiple sequence alignment in same page.

Multiple sequence alignment of protein sequences of each family was done using clustal X version 2.0. The phylogenetic tree of each family was generated using MEGA 4.1 (24) at default parameters with bootstrap value 1000 with Neighbor-Joining method.

#### Mutational profile

The mutational profile provides the information of all the possible residues at each position in each variant of a β-lactamase family. It was created using MEGA 4.1 tool. Mutation profile basically condenses the vast amount of mutational data into a readily interpretable form by showing the different/variable amino acids at each position of a β-lactamase family. Mutational profile differs from the variability plot by the fact that the former shows the amino acids present at a particular position whereas the later only tells extent of variability at a particular position.

## Data availability

The complete data content of CBMAR is free to download.

## Comparison with other available databases

In addition to the currently published repositories such as BLAD (14) and CARD (13), some previously reported β-lactamase databases contained manually curated and processed β-lactamase data. However, they do not provide any integration or detailed description for family-specific-studies, neither have they analyzed the molecular and biochemical data in a systematic manner. The advantage of CBMAR over other existing databases is that it provides information about various aspects of β-lactamase families like, genus/genera in which such family was reported, location on the genome, antibiotic resistance profile and susceptibility to β-lactamase inhibitor, active site, family-specific fingerprint, mutational profile and variability plot. A detailed comparison of CBMAR with other existing databases is shown in Figure 1. We hope the additional features of CBMAR can help in formulating a better-informed hypothesis, resulting in a better understanding of biochemical and physico-chemical consequences of mutations within different β-lactamases.

## Conclusions

The propagation of resistance in bacteria toward β-lactam antibiotics is a serious challenge for the scientific and pharmaceutical community. The active research done during the past has produced lots of data related to β-lactamase-mediated antibiotic resistance. Unfortunately, the accumulated data are spread among diverse and heterogeneous sources of information. We believe that the availability of a repository collecting and curating the β-lactamase variants would constitute a key resource for the scientific community. We expect that our database, CBMAR, will serve as an integrated data resource and help derive molecular and mechanistic details of β-lactamases. Further, CBMAR is also an analysis tool, which also contains information related to the β-lactamases and β-lactam hydrolysis that may aid the researchers in a comprehensive analysis of β-lactamase-mediated antibiotic resistance. We request the users to notify us of any additional new data so that we can maintain an updated CBMAR.

## Accessibility

The database and its content are freely accessible to all users without any restriction at http:/14.139.227.92/mkumar/lactamasedb.

## Supplementary data

Supplementary data are available at *Database* Online.

Supplementary Data
