# Supplementary material for: CBMAR: a comprehensive β-lactamase molecular annotation resource
Source: Database (Oxford). 2014 Dec 3;2014:bau111. doi: 10.1093/database/bau111 (PMC4255060; doi:10.1093/database/bau111)
Supplement: Supplementary Data [file supp_2014_bau111_index.html]

Supplementary Data 

# CBMAR: a comprehensive β-lactamase molecular annotation resource

## Supplementary Data

files

**Files in this Data Supplement:**

- Supplementary Data - zip file
