# Supplementary material for: CBMAR: a comprehensive β-lactamase molecular annotation resource
Source: Database (Oxford). 2014 Dec 3;2014:bau111. doi: 10.1093/database/bau111 (PMC4255060; doi:10.1093/database/bau111)
Supplement: Supplementary Data [file supp_bau111_suppl_data.zip › cbmar_supplementry_file.doc]

**Table S1.** Family-wise distribution of variants and proteins in each family of β-lactamase in CBMAR. Gram phenotype shows the character of pathogen carrying the β-lactamase gene. Genetic location indicates the gene location in genome.

| **Class** | **Family** | **Gram phenotype** | **Number of variants** | **Number of sequences in family** | **Genetic location** |
| --- | --- | --- | --- | --- | --- |
|  |  |  |  |  |  |
| A | AER | - | 1 | 1 | Plasmid |
|  | AST | + | 1 | 4 | Plasmid |
|  | BEL | - | 3 | 3 | Plasmid |
|  | BES | - | 1 | 1 | Plasmid |
|  | BLAF | + | 1 | 4 | Plasmid |
|  | BlaZ | + | 1 | 5 | Plasmid |
|  | BPS | - | 1 | 5 | Chromosomal |
|  | BRO | - | 2 | 17 | Chromosomal |
|  | CARB | - | 10 | 22 | Plasmid |
|  | CEPA | - | 6 | 7 | Chromosomal |
|  | CFXA | - | 6 | 10 | Chromosomal |
|  | CKO | - | 2 | 3 | Chromosomal |
|  | ClbA | - | 4 | 6 | Chromosomal |
|  | CME | - | 2 | 3 | Chromosomal |
|  | CSP | - | 1 | 1 | Chromosomal |
|  | CTXM | - | 127 | 214 | Plasmid |
|  | CumA | - | 1 | 1 | Chromosomal |
|  | DES | - | 1 | 1 | Chromosomal |
|  | FAR | + | 1 | 2 | Chromosomal/Plasmid |
|  | ERP | - | 1 | 1 | Chromosomal |
|  | FONA | - | 6 | 6 | Chromosomal/Plasmid |
|  | FEC-1 | - | 1 | 1 | Plasmid |
|  | GES | - | 21 | 33 | Plasmid |
|  | HUGA | - | 1 | 1 | Plasmid |
|  | HERA | - | 3 | 3 | Plasmid |
|  | IBC | - | 2 | 3 | Plasmid |
|  | IMI | - | 4 | 6 | Plasmid |
|  | BIC | - | 1 | 1 | Chromosomal |
|  | KLUA | - | 8 | 8 | Plasmid |
|  | KLUC | - | 3 | 3 | Plasmid |
|  | KLUG | - | 1 | 1 | Chromosomal |
|  | KLUY | - | 4 | 4 | Chromosomal |
|  | KPC | - | 17 | 97 | Plasmid |
|  | LAP | - | 2 | 4 | Plasmid |
|  | LEN | + | 7 | 16 | Chromosomal |
|  | LRG | - | 1 | 1 | Chromosomal |
|  | LUT | - | 6 | 6 | Chromosomal |
|  | MAL | - | 2 | 2 | Chromosomal |
|  | NMC | - | 1 | 2 | Chromosomal |
|  | OHIO | - | 1 | 1 | Plasmid |
|  | OKP | - | 2 | 25 | Chromosomal |
|  | ORN | - | 6 | 6 | Chromosomal |
|  | OXY | - | 14 | 27 | Chromosomal |
|  | PENA | - | 1 | 18 | Chromosomal |
|  | PER | - | 7 | 17 | Plasmid |
|  | PLA | - | 4 | 4 | Chromosomal |
|  | PSE | - | 5 | 46 | Plasmid |
|  | RAHN | - | 2 | 2 | Chromosomal |
|  | RTG | - | 2 | 2 | Plasmid |
|  | ROB | + | 1 | 11 | Plasmid |
|  | SCO | - | 1 | 5 | Plasmid |
|  | SED | - | 1 | 1 | Chromosomal |
|  | SFC | - | 1 | 1 | Plasmid |
|  | SFO | - | 1 | 2 | Chromosomal/Plasmid |
|  | SHV | - | 140 | 221 | Plasmid/Chromosomal |
|  | SME | - | 5 | 6 | Chromosomal |
|  | TEM | - | 148 | 320 | Plasmid |
|  | TER | - | 2 | 2 | Chromosomal |
|  | TLA | - | 2 | 2 | Plasmid |
|  | TOHO | - | 2 | 4 | Plasmid |
|  | VHH | - | 1 | 1 | Plasmid |
|  | VHW | - | 1 | 1 | Plasmid |
|  | VEB | - | 8 | 20 | Plasmid |
|  | ACI | - | 1 | 1 | Plasmid |
| B1 | Blab | - | 14 | 23 | Chromosomal |
|  | CcrA | - | 1 | 1 | Plasmid |
|  | CGB | - | 1 | 1 | Plasmid |
|  | DIM | - | 1 | 2 | Integron |
|  | EBR | - | 1 | 1 | Chromosomal |
|  | GIM | - | 1 | 7 | Plasmid |
|  | IMP | - | 39 | 126 | Plasmid/Chromosomal |
|  | IND | - | 14 | 19 | Chromosomal |
|  | JOHN | - | 1 | 1 | Chromosomal |
|  | KHM | - | 1 | 1 | Plasmid |
|  | NDM | - | 10 | 32 | Plasmid |
|  | SFB | - | 1 | 1 | Chromosomal |
|  | SIM | - | 1 | 3 | Plasmid |
|  | SLB | - | 1 | 1 | Chromosomal |
|  | SPM | - | 1 | 1 | Chromosomal |
|  | TMB | - | 1 | 2 | Chromosomal |
|  | TUS | - | 1 | 1 | Chromosomal |
|  | MUS | - | 1 | 1 | Chromosomal |
|  | VIM | - | 34 | 79 | Plasmid |
| B2 | CphA | - | 7 | 12 | Chromosomal |
|  | SFH | - | 1 | 1 | Chromosomal |
|  | ImiS | - | 1 | 1 | Unknown |
| B3 | AIM | - | 1 | 1 | Chromosomal |
|  | AMO | - | 1 | 1 | Chromosomal |
|  | L1 | - | 1 | 20 | Chromosomal |
|  | BJP | - | 1 | 1 | Unknown |
|  | CAU | - | 1 | 1 | Chromosomal |
|  | FEZ | - | 1 | 1 | Chromosomal |
|  | GOB | - | 10 | 12 | Chromosomal |
|  | LRA | - | 12 | 12 | Unknown |
|  | M1B1B | - | 1 | 1 | Unknown |
|  | POM-1 | - | 1 | 1 | Chromosomal |
|  | SMB | - | 1 | 1 | Plasmid |
|  | THIN-B | - | 1 | 1 | Plasmid |
| C | ACC | - | 10 | 12 | Chromosomal |
|  | AmpC | - | 1 | 156 | Plasmid |
|  | ACT | - | 14 | 14 | Plasmid |
|  | BIL | - | 1 | 1 | Plasmid |
|  | CFE | - | 1 | 1 | Plasmid |
|  | CMY | - | 89 | 112 | Plasmid |
|  | DHA | - | 7 | 15 | Plasmid |
|  | EBC | - | 1 | 1 | Plasmid |
|  | FOX | - | 10 | 12 | Plasmid |
|  | LAT | - | 3 | 3 | Plasmid |
|  | MIR | - | 5 | 6 | Plasmid |
|  | MOX | - | 7 | 7 | Plasmid |
|  | SRT | - | 1 | 2 | Chromosomal |
|  | SST | - | 1 | 1 | Chromosomal |
| D | OXA | - | 242 | 363 | Plasmid |
|  | LCR | - | 1 | 5 | Plasmid |
