# Supplementary material for: CBMAR: a comprehensive β-lactamase molecular annotation resource
Source: Database (Oxford). 2014 Dec 3;2014:bau111. doi: 10.1093/database/bau111 (PMC4255060; doi:10.1093/database/bau111)
Supplement: Supplementary Data [file supp_bau111_suppl_data.zip › New Microsoft Office Word Document.docx]

# Supplementary Data

**Table S1:** Family-wise distribution of variants and proteins in each family of β-lactamase in CBMAR.
